# Targeted metatranscriptomics of compost-derived consortia reveals a GH11 exerting an unusual exo-1,4-β-xylanase activity

**DOI:** 10.1186/s13068-017-0944-4

**Published:** 2017-11-02

**Authors:** Bruno L. Mello, Anna M. Alessi, Diego M. Riaño-Pachón, Eduardo R. deAzevedo, Francisco E. G. Guimarães, Melissa C. Espirito Santo, Simon McQueen-Mason, Neil C. Bruce, Igor Polikarpov

**Affiliations:** 1 Instituto de Física de São Carlos, Universidade de São Paulo, Av. Trabalhador São-carlense 400, São Carlos, SP 13560-970 Brazil; 2 Department of Biology, University of York, Wentworth Way, York, YO10 5DD UK; 3 Laboratório Nacional de Ciência e Tecnologia do Bioetanol, Centro Nacional de Pesquisa em Energia e Materiais, Rua Giuseppe Máximo Scalfaro 10000, Campinas, SP 13083-100 Brazil; 4 Laboratório de Biologia de Sistemas Regulatórios, Departamento de Química, Instituto de Química, Universidade de São Paulo, Av. Prof. Lineu Prestes 748, São Paulo, SP 05508-000 Brazil

**Keywords:** Metatranscriptomics, Xylanase, Lignocellulose, Compost, Microbial community, Bioethanol

## Abstract

**Background:**

Using globally abundant crop residues as a carbon source for energy generation and renewable chemicals production stand out as a promising solution to reduce current dependency on fossil fuels. In nature, such as in compost habitats, microbial communities efficiently degrade the available plant biomass using a diverse set of synergistic enzymes. However, deconstruction of lignocellulose remains a challenge for industry due to recalcitrant nature of the substrate and the inefficiency of the enzyme systems available, making the economic production of lignocellulosic biofuels difficult. Metatranscriptomic studies of microbial communities can unveil the metabolic functions employed by lignocellulolytic consortia and identify novel biocatalysts that could improve industrial lignocellulose conversion.

**Results:**

In this study, a microbial community from compost was grown in minimal medium with sugarcane bagasse sugarcane bagasse as the sole carbon source. Solid-state nuclear magnetic resonance was used to monitor lignocellulose degradation; analysis of metatranscriptomic data led to the selection and functional characterization of several target genes, revealing the first glycoside hydrolase from Carbohydrate Active Enzyme family 11 with exo-1,4-β-xylanase activity. The xylanase crystal structure was resolved at 1.76 Å revealing the structural basis of exo-xylanase activity. Supplementation of a commercial cellulolytic enzyme cocktail with the xylanase showed improvement in Avicel hydrolysis in the presence of inhibitory xylooligomers.

**Conclusions:**

This study demonstrated that composting microbiomes continue to be an excellent source of biotechnologically important enzymes by unveiling the diversity of enzymes involved in in situ lignocellulose degradation.

**Electronic supplementary material:**

The online version of this article (10.1186/s13068-017-0944-4) contains supplementary material, which is available to authorized users.

## Background

The accelerated rate of fossil fuel depletion and concerns over global warming has triggered the search for renewable energy sources. Lignocellulose is the basic component of plant cell walls and one of the most abundant sources of carbon in the biosphere. Therefore, its bioconversion into liquid fuels represents a promising solution for energy generation [1–3].

In recent years, direct DNA extraction techniques from microbial communities coupled with next generation sequencing of metagenomes have given an unprecedented insight into microbial taxonomic groups and their interactions [4]. Metagenomic libraries also represent a vast resource for the discovery of enzymes with industrial applications.

Although many free-living organisms deconstruct plant biomass by enzyme-driven oxidation and hydrolysis [5], this bioprocess remains a formidable challenge for industry. One of the main obstacles to industrial-scale production of second-generation biofuel lies in the inefficient deconstruction of plant material, due to the recalcitrant nature of the substrate and relatively low activity of currently available enzymes [6].

Seeking to overcome these challenges, previous studies have sequenced and functionally characterized microbial communities from different biomass-degrading environments. Examples include microbial communities from compost [7–9], bovine rumen [6, 10], guts of animals [11–16], soil [17, 18], and river water [19]. These studies have revealed the lignocellulolytic capabilities of microbial communities present in diverse ecosystems and the highly complex and cooperative interactions between multiple microbial species and their enzymes to achieve lignocellulose breakdown.

In this study, a compost-derived microbial community was grown in minimal medium supplemented with sugarcane bagasse as a sole carbon source aiming to enrich lignocellulose-degrading microorganisms. We monitored deconstruction of sugarcane bagasse using scanning electron microscopy, solid-state nuclear magnetic resonance (ssNMR) spectroscopy and confocal microscopy. To obtain information on the community response to this submerged in vitro environment, metatranscriptomic analysis was performed. A number of predicted genes that showed similarity to carbohydrate active enzymes (CAZymes) were selected for expression leading to the discovery of the first exo-1,4-β-xylanase from glycoside hydrolase family 11 (GH11). This enzyme was able to degrade xylooligomers, which are known inhibitors to commercially available cellulase cocktails [20], as well as xylan, yielding xylobiose as the only reaction product.

## Results

### Compositional and morphological changes in sugarcane bagasse

The compositional analysis of sugarcane bagasse collected weekly from in vitro composting cultures was investigated using ssNMR. In order to obtain ^13^C quantitative spectra in an achievable measuring time, the spectra were acquired using the multiple cross polarization pulse sequence (Multi-CP) under fast (14 kHz) Magic Angle Spinning [21]. Using Multi-CP, the integral of each signal in the NMR spectra is proportional to the amount of the corresponding chemical group in the sample. Therefore, quantitative information on sample relative composition can be obtained if a reliable identification of the NMR lines is available [21, 22]. The complete assignment of the signals can be found in references [23–31].

**Fig. 1:**
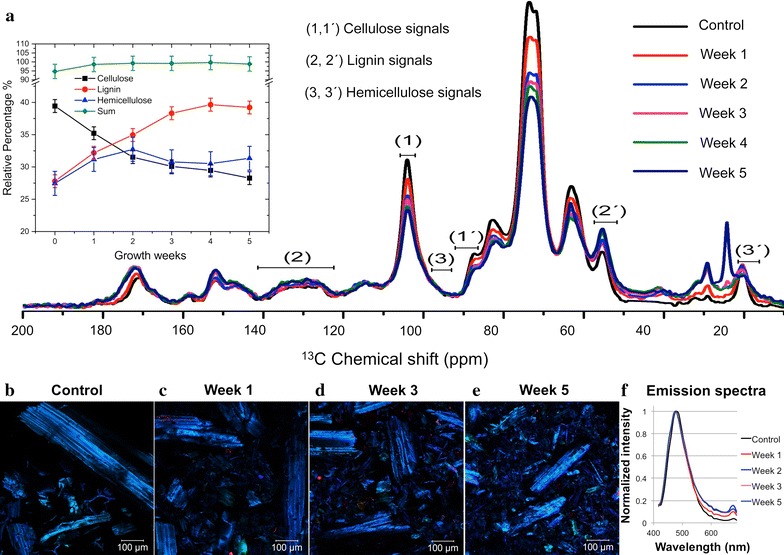
Analysis of sugarcane bagasse composition. **a** Solid-state nuclear magnetic resonance spectra of sugarcane bagasse prior and after microbial growth. Regions 1 and 1′ are assigned to the C1 carbon of cellulose (103–106 ppm) and to C4 carbon of crystalline cellulose (86–92 ppm). Regions 2 and 2′ are assigned to C1, C2, and C4 aromatic carbons of lignin (123–142 ppm) and to aryl methoxyl carbons of lignin (50–56 ppm). Regions 3 and 3′ are assigned to C1 carbon of hemicellulose and to CH_3_ in acetyl groups of hemicelluloses. The relative abundance of cellulose, hemicellulose, and lignin was estimated from regions 1, 1′, 2, 2′, 3, and 3′. **b**–**e** Confocal images of sugarcane bagasse lignin prior and after microbial growth. **f** Corresponding average emission spectra

Figure 1a shows the sugarcane bagasse ^13^C Multi-CP spectra with spectral regions specifically assigned to three major lignocellulose components (cellulose, hemicellulose, and lignin) highlighted. After normalizing the spectra by the total area, we used the integrals over the specified regions to estimate the cellulose, hemicellulose, and lignin fractions in the sample [32]. The plot of this relative percentage is depicted as a function of the growth weeks. Due to microbial growth and enzymatic activities of the composting cultures, a gradual reduction in the relative amount of cellulose was observed. The relative percentage of hemicellulose remained mostly constant, with some fluctuation attributed to experimental uncertainties. Consistently, the relative amount of lignin increased at the same rate that cellulose decreased.

Lignin concentration and arrangement before and after microbial growth were investigated by confocal imaging microscopy using two-photon excitation [33]. The analysis showed no change in the emission spectra after 5 weeks of microbial growth (Fig. 1b–f). This observation supports the ssNMR results that our compost microbial communities promoted insignificant lignin degradation. It also suggests that the structure or organization of the lignin residues remained mostly unmodified.

**Fig. 2:**
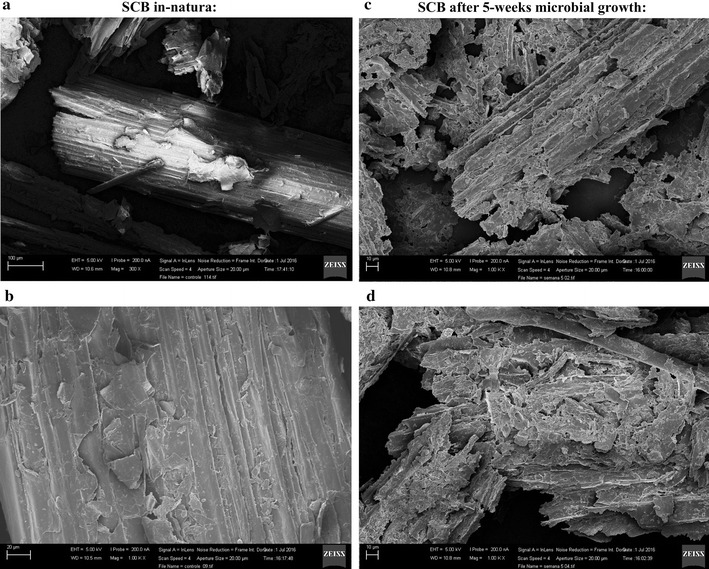
Scanning electron microscopy imaging of the sugarcane bagasse prior **a**, **b** and after 5 weeks **c**, **d** of microbial growth analyzed in × 2 magnifications

Further, we obtained scanning electron microscopy images of the sugarcane bagasse before and after 5 weeks of microbial growth. Control biomass sample (no inoculum) showed a smooth, continuous surface with cohesive, well-defined lignocellulose fibers (Fig. 2). In contrast, five weeks of microbial growth caused a complete loss in the biomass integrity, with separation of the fibers and decrease of particles’ size.

### Functional and phylogenetic characteristics of the sugarcane bagasse degrading microbial community

**Fig. 3:**
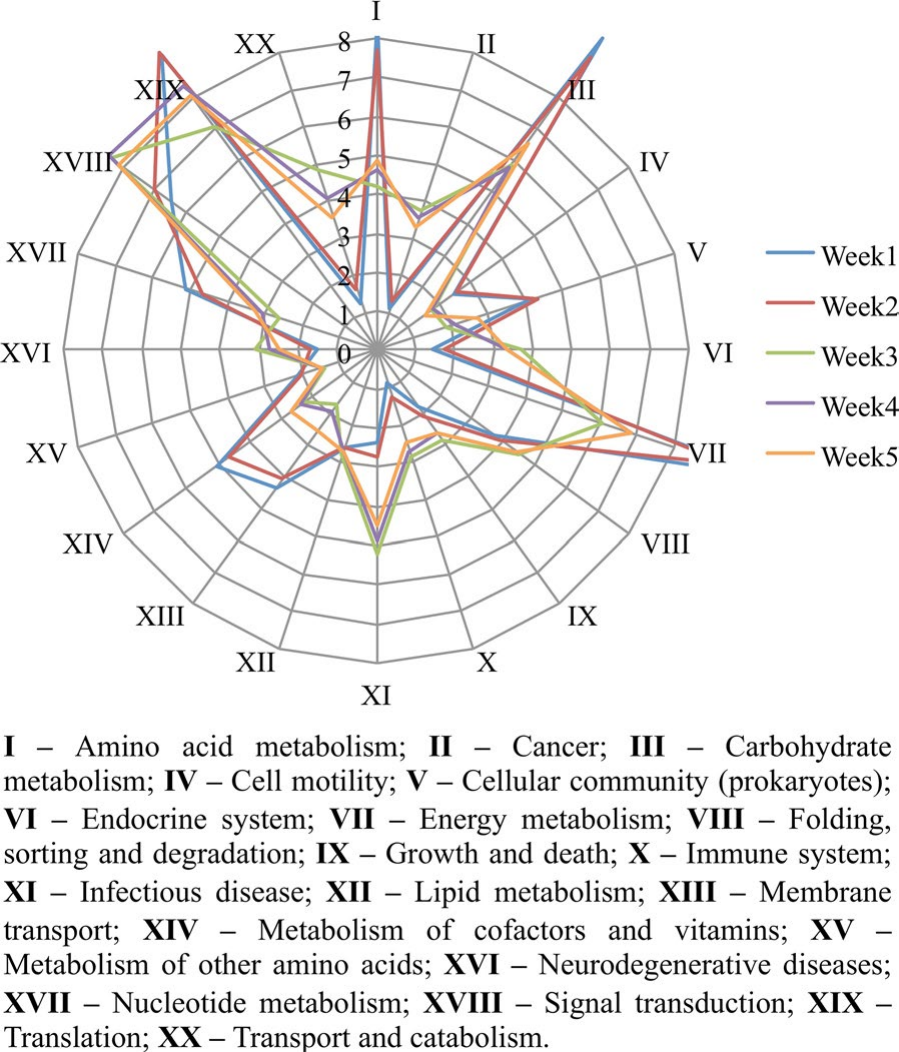
Functional profile of KEGG-assigned genes in sugarcane bagasse composting community metatranscriptome. Relative abundance of predicted open reading frames in terms of the KEGG function was assigned. Value for a functional profiles is normalized by the sum of all functions for each time point

To examine the transcriptional responses of the sugarcane bagasse degrading microbial community, we performed RNA-seq metatranscriptomic analysis on weekly cultures grown for up to 5 weeks. Although it yielded 66 million paired-end reads (Table 1), the rarefaction analysis showed that the sequencing did not reach saturation (Additional file 1: Figure S1). The rarefaction analysis also revealed that week 1 cultures were more diverse than week 5 cultures since a larger proportion of new reads was obtained at the same sequencing depth. Resulting high-quality, non-ribosomal RNA reads (63%) were de novo assembled into 302,961 transcripts and used to predict biochemical capabilities of the microbial community by mapping putative transcripts to the KEGG orthology. The distribution of genes classified to KEGG functions presented a similar profile for all time points (Fig. 3). Transcripts assigned to translation processes (mean = 8.5%) showed the highest relative abundance, followed by genes involved in energy, carbohydrate, and amino acids metabolism and signal transduction (mean from 6 to 8%). We observed that the microbial community was more actively growing and breaking down the lignocellulose in initial stages of culture since a number of transcripts assigned to energy and carbohydrate metabolism were higher in week 1 compared to week 5 cultures.

**Table 1:** Metatranscriptome sequencing and processing metrics

	Week 1	Week 2	Week 3	Week 4	Week 5	Total
Total reads generated	20,119,184	14,715,430	7,767,801	5,879,612	17,825,076	66,307,103
Reads after quality filtering	15,916,667	12,072,389	6,757,570	5,111,343	14,535,407	54,393,376
mRNA reads	12,645,050	10,652,024	3,608,818	3,971,178	11,004,487	41,881,555
Assembled transcripts	104,408	64,923	27,821	34,023	71,786	302,961
N50	947	888	789	817	858	912
Average size	963	921	820	847	884	887
Predicted open reading frames	104,425	59,885	23,156	31,198	64,692	283,356
Lignocellulose active	3012	1523	534	654	1473	7196

**Fig. 4:**
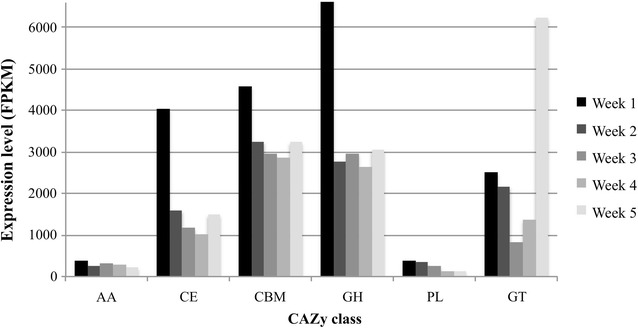
Expression of putative CAZymes in the microbial community metatranscriptome. *AA* auxiliary activities, *CE* carbohydrate esterases, *CBM* carbohydrate binding modules, *GH* glycoside hydrolases, *GT* glycosyl transferases, *PL* polysaccharide lyases

Next, the metatranscriptome libraries were screened using HMMER alignment tool [34] and the dbCAN database [35] for genes encoding putative CAZymes involved in lignocellulose degradation. From the 283,356 predicted open reading frames (ORFs), 2.5% (number of sequences = 7196) showed homology to CAZymes. The CAZymes were distributed between carbohydrate binding modules (34.4%), glycoside hydrolases (34.3%), glycosyl transferases (19.3%), carbohydrate esterases (15.9%), auxiliary activities (3.3%), polysaccharide lyases (2.2%), and cohesin and dockerin modules (0.8%). The expression level for the majority of CAZyme classes was higher in week 1 cultures (Fig. 4). KEGG-assigned transcripts involved in carbohydrate metabolism presented a similar profile. Transcripts predicted as glycosyl transferases showed contrasting behavior with the highest expression level at week 5.

**Fig. 5:**
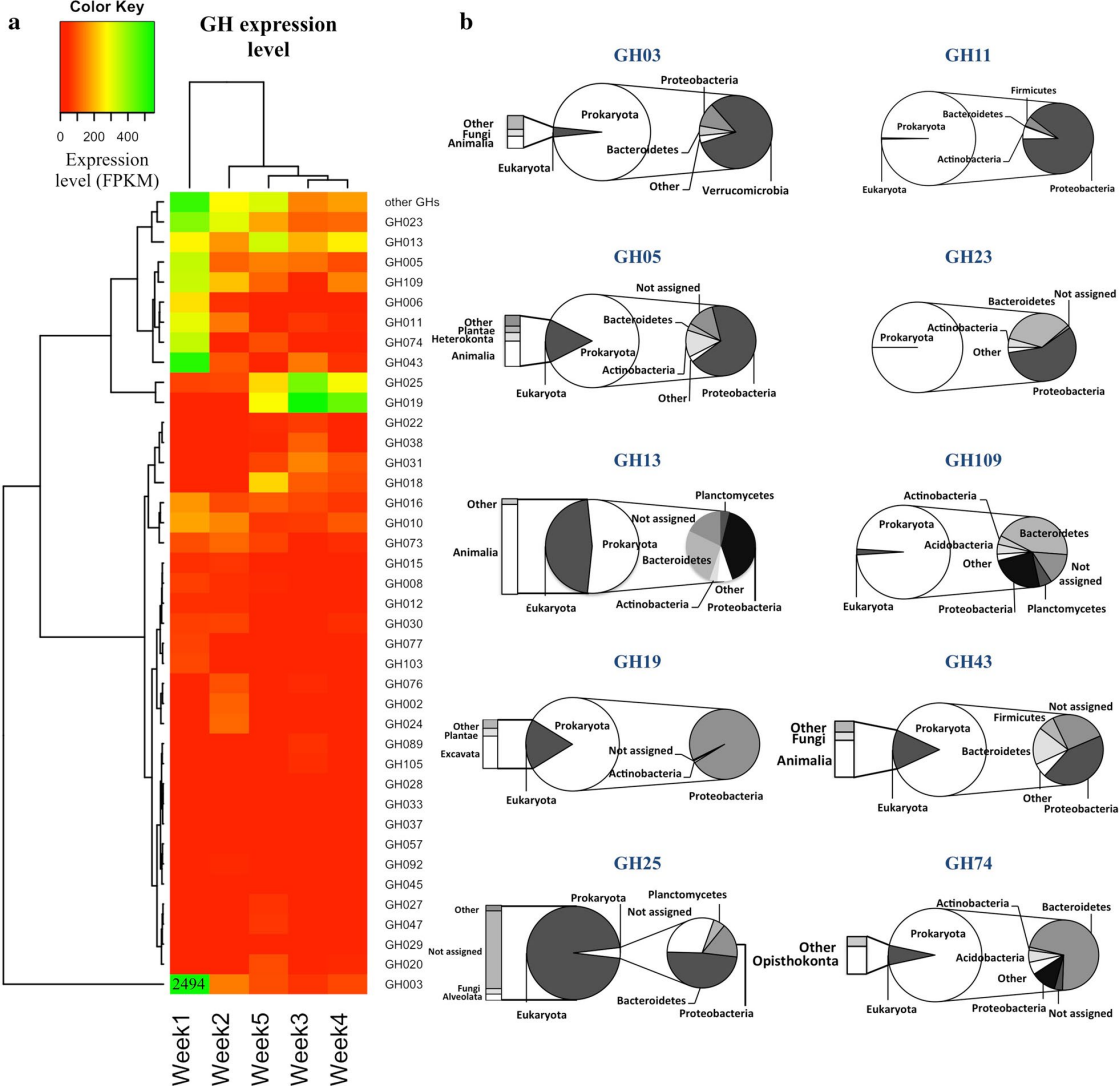
Differential expression and phylogenetic distribution of glycoside hydrolase (GH) families identified in sugarcane bagasse composting community metatranscriptome. **a** Heat map representation of the GHs expression. Columns represent time when sample was collected. Rows depict different GHs families identified in the metatranscriptome dataset. The color key for GH 3 expression at week 1 is out of range with expression level of 2494. GH families were grouped based on substrate preferences, as reported elsewhere [93]. **b** Phylogenetic assignment of reads belonging to the most expressed families using the Lowest Common Ancestor algorithm

Since the most expressed CAZy-assigned transcripts were predicted as glycoside hydrolases (GHs), we analyzed the distribution and phylogenetic origin of these ORFs in details (Fig. 5). Endoglucanases (GH5, GH74) acting on the cellulose backbone and β-glucanases (GH3) involved in cellobiose hydrolysis, showed high expression at week 1, followed by a gradual decline over time (Fig. 5a). Similar profile was observed for endoxylanases (GH10, GH11) and hemicellulose debranching enzymes such as arabinofuranosidases (GH43). On the contrary, annotated lysozymes and chitinases from GH19 and GH25 families displayed higher expression in the later time points, indicating that these cell wall lytic enzymes might be associated with an increase of competitive interactions between microbial species in the later stages of culture. Phylogenetic origin of predicted GHs was also investigated (Fig. 5b). Proteobacteria expressed the majority of GH5 endoglucanases, GH11 endoxylanases, and GH19 lysozymes, whereas Bacteroidetes members were major producers of GH13 amylases, GH23 lysozymes, GH43 hemicellulose debranching enzymes, and GH109 α-*N*-acetylgalactosaminidase. The GH3-assigned transcripts were mostly expressed by species of Verrucomicrobia phylum. Some GH families were also predicted to derive from eukaryotes. Specifically, starch and glycogen degrading enzymes of GH13 family were highly expressed by eukaryotes from the Animalia kingdom. However, majority of GH25 lysozymes were not assigned beyond Domain level. The distribution of prokaryotic and eukaryotic origin of CAZymes (all classes) was further examined by the relative expression level of predicted enzymes assigned to specific phylogenetic level. Among the 7196 predicted CAZyme genes, 75% were taxonomically assigned to phylum level. It revealed that the microorganisms most actively involved in carbohydrate modification belonged to Bacteroidetes and Proteobacteria (Additional file 1: Figure S2). Those lineages accounted for more than 65% of the bacterial diversity over all time points. The expression of CAZymes affiliated to Bacteria dropped from 20,171 to 6465 fragments per kilobase of transcript per million (FPKM) over the five-week time course. During this period, genes encoding putative CAZymes of eukaryotic origin showed a dramatic increase by 30-fold. At week 5, 82% of genes expressed by Eukaryotes were assigned to the kingdom Animalia with only 3% to be predicted as fungal genes.

In order to quantify the relative percentage of bacteria to fungi in the sugarcane bagasse degrading community, real-time PCR was performed. Note two differences with the results described in the above paragraph: the phylogeny is regarding the entire community, not only CAZymes; the primers used to capture the Eukaryotic component of the community are specific to fungi. Overall Bacteria dominated the composting community (Additional file 1: Table S1) but the fungal/bacterial ratio in weekly samples gradually increased from 5 to 20% by the 3rd week and stabilized in older cultures.

### Heterologous expression and characterization of putative CAZymes

Following metatranscriptome analysis, we selected 27 GH assigned transcripts with predicted cellobiohydrolase, endoglucanase, and xylanase activities and higher expression levels for functional characterization (Additional file 1: Table S2). Following initial recombinant expression screening, seven out of twenty seven proteins (26% efficiency) were obtained in the soluble fraction of *E. coli* transformed cultures of which three proteins named compost7_GH6, compost13_GH10, and compost21_GH11 showed an enzymatic activity after assaying against a variety of polysaccharide substrates (Additional file 1: Figure S3).

BLASTP results against the NCBI-nr database showed that the protein compost7_GH6 has 49% identity to a GH6 β-1,4-glucan cellobiohydrolase from *Sorangium cellulosum*. Substrate screening showed that compost7_GH6 had activity against β-glucan and lichenan. No enzymatic activity was detected towards filter paper and carboxymethyl cellulose (CMC). Compost7_GH6 displayed highest activity towards β-glucan at pH 10.0 and was able to retain 70% or more activity until the pH dropped to 4.0 (Additional file 1: Figure S3). Thermal shift assays (see “Methods”, Additional file 1: Figure S4) confirmed the alkaliphilic behavior of the enzyme with the highest thermostability at pH 6 to 9. The optimal temperature was assayed at both pH 6.0, where the enzyme was found to maintain > 70% maximum activity, and 10.0. The highest activity at pH 6.0 and 10.0 was found at 50 and 45 °C, respectively. Next, the optimal pH and temperature were used to test enzyme specificity. The results showed that compost7_GH6 had highest specific activity towards β-glucan (2.0 U/mg) and lichenan (1.5 U/mg) amongst the substrates tested (Additional file 1: Figure S3c). We also determined that compost7_GH6 maintained 50% of its initial activity after 24 h incubation at 45 °C at pH 6.0 and 100% activity after 96-h incubation at 40 °C at pH 10.0.

The protein C13 was predicted as a member of GH10 family and had 91% and 89% identity to an endoglucanase and endo-β-1,4-xylanase from *S. cellulosum*, respectively. C13 showed endoxylanase activity against xylan, which was subsequently used as a substrate to determine the enzyme’s optimal pH and temperature. C13 displayed highest activity at pH 6.0 and retained > 50% of its activity over a broad pH range (pH 3.0–10.0) (Additional file 1: Figure S3c). The optimum temperature for enzyme activity was 65 °C at pH 6.0. These conditions were subsequently applied to test the enzyme specificity. The highest specific activity was found for xylan (25 U/mg) and arabinoxylan (11 U/mg). No activity was found against CMC. The residual activity study performed at 50 °C, pH 6.0 revealed that the enzyme retains more than 60% of its initial activity up to 96 h of incubation, demonstrating considerable thermal stability (Additional file 1: Figure S4).

The protein compost21_GH11 was predicted as a GH11 family member and shared 77% identity with a non-characterized GH from *Marinimicrobium agarilyticum*. The fully characterized homologue of compost21_GH11, was a β-1,4-xylanase from *S. cellulosum*, with 40% identity. Compost21_GH11 was found to be an exo-1,4-β-xylanase with highest activity against xylan at pH 6.0 at 35 °C. The enzyme retained more than 60% activity for all tested pHs, but the observed activity quickly dropped at temperatures higher than 40 °C. However, at 35 °C and pH 6.0, compost21_GH11 retained 90% activity for up to 96 h. The activity screen against a number of polysaccharides revealed that compost21_GH11 was active towards xylan only. Using this substrate, compost21_GH11 showed a high specific activity of 320 U/mg even at the relatively low reaction temperature (35 °C).

### Characterization and structure of compost21_GH11

**Fig. 6:**
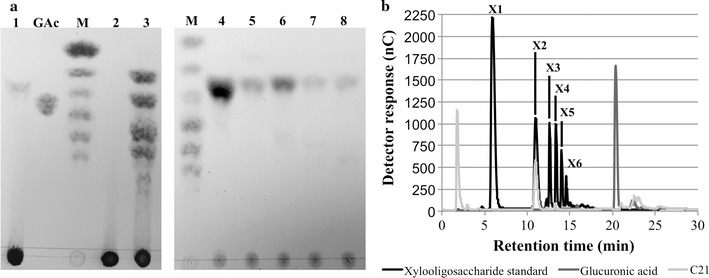
Experimental evidence that compost21_GH11 is an exo-1,4-β-xylanase. **a** Thin layer chromatography (TLC) of the products formed by: lanes 1 and 3: compost21_GH11 and compost13_GH10 action on xylan, respectively; lane 2: reaction blank; lanes 4–8: compost21_GH11 action on *X*2 to *X*6; *GAc* glucuronic acid; M: *X*1–*X*6 standard. **b** HPAEC-PAD of the products formed by compost21_GH11 action on xylan

Since compost21_GH11 presented no activity against substrates with a xylan backbone such as AZCL-linked xylan, a substrate specific for endoxylanases due to its cross-linked structure and dye labels [36], or arabinoxylan, this protein was chosen as a target for further characterization. To investigate the mode of action of compost21_GH11, we analyzed the reaction products by thin layer chromatography (TLC) and Dionex HPLC (Fig. 6). TLC results showed that compost21_GH11 was acting on xylan and xylooligosaccharides liberating xylobiose as the only reaction product. Dionex HPLC confirmed this hydrolysis pattern. Testing the enzyme with 4-nitrophenyl-β-d-xylopyranoside displayed no activity, confirming that the enzyme was not able to hydrolyze xylobiose.

**Fig. 7:**
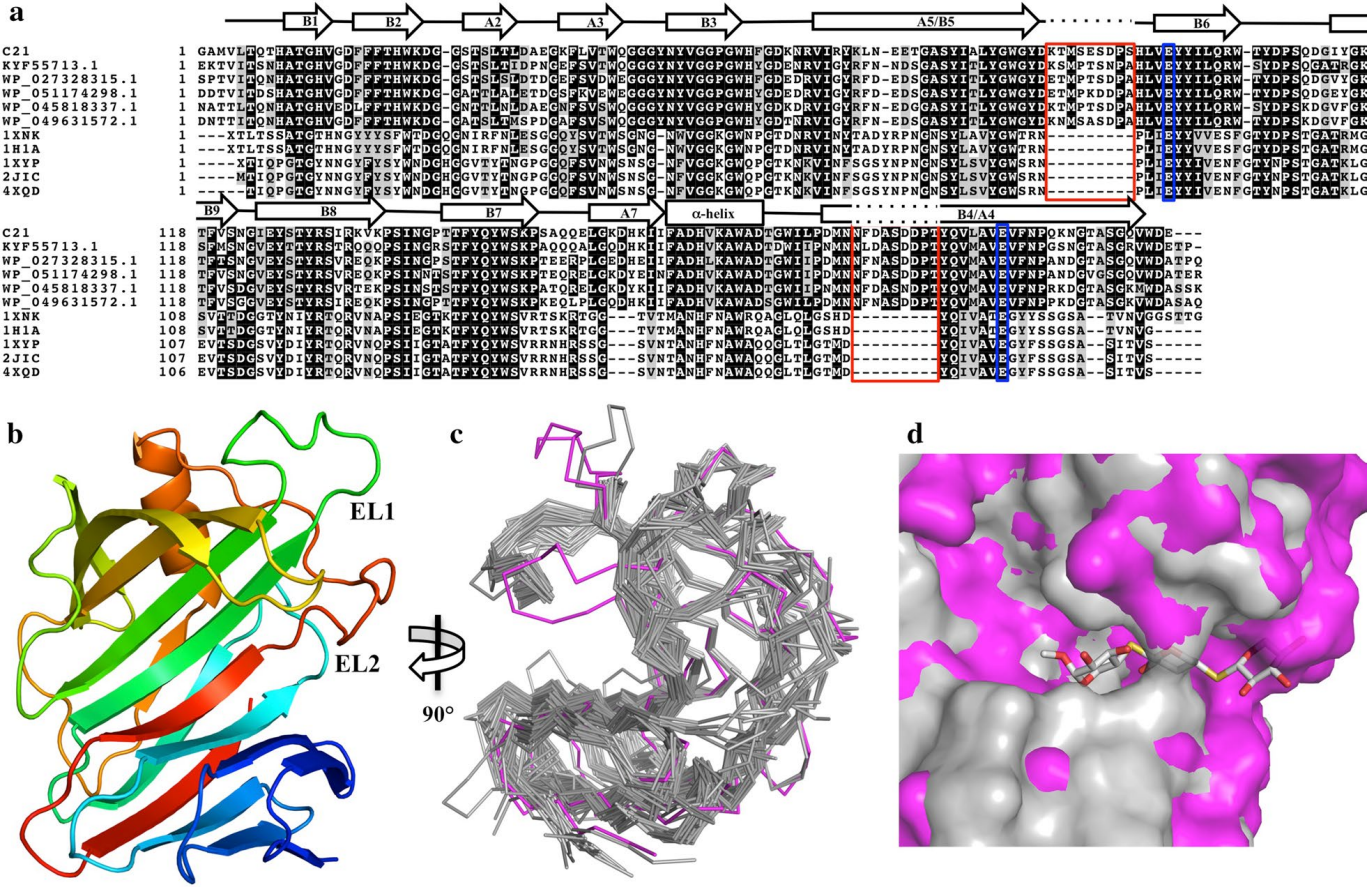
Structural evidence that compost21_GH11 is an exo-1,4-β-xylanase. **a** Amino acid sequence multiple alignment of compost21_GH11 with closest homologues selected based on searches in the NCBI-nr and PDB databases. The residues responsible for formation of extra loops 1 and 2 (EL1 and EL2) are shown in a red box while the catalytic residues are marked in a blue box. The extra loops are present in other proteins for which structure has not been solved. **b** Overall secondary structure of compost21_GH11 colored from blue to red (N- to C-terminal). **c** Superposition of all available GH11 structures (in gray) with compost21_GH11 (in magenta). The orientation of the structures is rotated by 90° in relation to the structure presented on item B. The non-aligned domains from proteins with non-common domains were hidden from representation. **d** Surface representation of compost21_GH11 (in magenta) aligned to closest PDB homologue 1XNK (in gray). Note that the non-reducing end of the ligand methyl 4,4^II^-dithio-*α*-xylotrioside present in 1XNK structure is sterically hindered by EL2 in compost21_GH11 − 3 subsite

In order to understand the molecular basis of exo-1,4-β-xylanase activity, the crystal structure of compost21_GH11 was solved at 1.76 Å resolution. The data collection and refinement statistics are summarized in Table 2. The final model was constructed from the first to last residue of the crystallized protein, which was cloned without the first 13 residues that were predicted as a disordered region and reported as a signal peptide [37]. Despite its low identity to the closest homologue in PDB (ID 1XNK, 36% identity), compost21_GH11 presents a typical GH11 fold. It consists of one α-helix and 15 β-sheets, labeled A1 to A6 and B1 to B9 (Fig. 7a). The curvature of the β-sheets B1 to B9 forms a cleft where the catalytic residues (nucleophile Glu98 in β-sheet B6 and proton donor Glu200 in β-sheet B4) are located. Two extra loops (EL1 and EL2) are found in the compost21_GH11 structure. They are created by additional residues, which stretch between β-sheets B5 and B6 and in β-sheet B4, respectively. To gain insight into substrate binding, 1XNK, which was co-crystallized with a modified xylotrioside, was aligned to compost21_GH11 (root mean square deviation of 0.898 Å when EL1 and 2 are ignored). EL2 blocks substrate interaction at subsite − 3, which accommodates the non-reducing end of xylan (Fig. 7d). This loop is stabilized by a number of hydrogen bonds within the loop main/side chain residues and van der Walls interactions, especially between Pro192 and Phe186 side chains. Hydrogen bonds are also established with EL1 and with the turn that connects β-sheets B7 and B8. The presence of EL1 seems essential to maintain EL2 in correct position by steric hindrance.

**Table 2:** Data collection and refinement statistics of compost21_GH11 structure

Data collection	
Wavelength/beamline	1.45866/MX2, LNLS
Space group	P4_3_2_1_2
Unit cell dimensions (Å)	64.33; 64.33; 105.87
Molecules/asymmetry unit	1
Matthews coefficient (Å^3^/Da)	2.24
Solvent content (%)	45.0
Resolution (Å)	1.76
Number of unique reflections	22,638 (1226)
Mosaicity (°)	0.205
Multiplicity	24.0
Completeness	99.6
Refinement	
Number of amino acid residues	216
Number of waters	282
*R*_work_/*R*_free_ (%)	19.4/21,8
RMS bond lengths (Å)	0.069
RMSD bond angles (°)	1.144
Mean overall B-factor (Å^2^)	19.5
Ramachandran in most favored regions (%)	96.73
Ramachandran outliers (%)	0
PDB ID	5VQJ

Values in parenthesis refer to the outer shell. *R*_free_ was calculated with 5% of the reflections that were randomly chosen and excluded from the refinement

### Compost21_GH11 activity improves performance of a commercial enzyme mixture

**Fig. 8:**
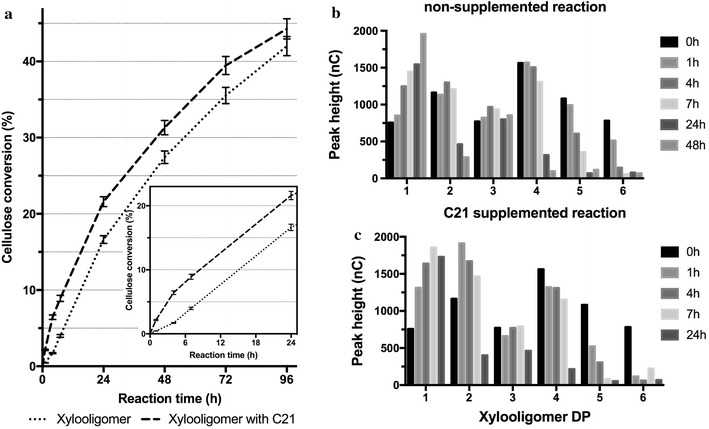
Impact of xylooligomers on commercial cocktail performance. **a** Hydrolysis of Avicel by Accellerase^®^ in the presence of xylooligomers and compost21_GH11 during 96-h time course. **b** HPLC analysis showing xylooligomer degradation by Accellerase^®^ and **c** Accellerase^®^ supplemented with compost21_GH11, as assessed by detector response in nanocoulombs (nC). Note that in Fig. 3c xylobiose concentration increases after 1 h as a result of fast degradation of xylooligomers with degree of polymerization (DP) ≥ 4 by compost21_GH11. Higher DP xylooligomers have a higher inhibitory effect, thus its rapid degradation seems beneficial. The commercial cocktail used was able to degrade the added xylooligomers within 24 h. In consequence, after 96 h, reactions with and without compost21_GH11 achieved about the same glucose yields. However, at initial stages, the addition of compost21_GH11 strongly improved glucose production rate

To examine the effect of xylooligomers on cellulose digestion, we monitored the hydrolysis of 2% (w/v) Avicel by a commercial enzymatic cocktail in the presence or absence of oligosaccharides. The reactions improvements were further tested by supplementing the reactions with compost21_GH11 protein. Figure 8a shows that xylooligomers strongly inhibited Accellerase activity, especially at the initial time points. The addition of xylooligomers resulted in activity decrease of 96.5% at 1 h; after 96 h, the activity decrease was 45%. This indicates that the enzymes present in the commercial cocktail were able to degrade, to some extent, the xylooligomers, reducing their inhibitory effect. When Accellerase was supplemented with compost21_GH11, the addition of xylooligomers decreased the activity by 83% at 1 h; after 96 h, the activity decrease was 53%. Hence, inhibition was much lower, particularly at initial stages (where a 380 times difference is seen between compost21_GH11 supplemented and non-supplemented reactions). Dionex HPLC demonstrated that xylooligomers with polymerization degree higher than 4 were depleted from compost21_GH11 non-supplemented reactions after 24 h, whereas it took only 4 h for the xylooligomers to be depleted when supplemented with compost21_GH11. Since the xylooligomer concentration was reduced in both reactions, they reached approximately the same conversion after 96 h.

## Discussion

Plant cell walls are effectively degraded in various natural ecosystems by the action of microorganisms that act cooperatively by secreting an array of lignocellulolytic enzymes. In recent years, metatranscriptomic analysis applied to these ecosystems has begun to provide an insight into how lignocellulose breakdown is accomplished in situ [2, 38, 39].

Here, we investigated the time course degradation of sugarcane bagasse by a microbial community derived from compost. Based on sugarcane bagasse biomass analysis, we showed that the lignin component remained mostly unchanged and was not significantly modified by microbial activities. Our analysis was in agreement to a previous study [1] showing that biomass loss is mostly attributed to cellulose and hemicellulose degradation. Despite this limitation, composting community remained metabolically active during the experiment as surveyed by RNA sequencing.

Next, we explored the metatranscriptome-assembled library, by focusing on screening the resulting database for CAZymes. Although the predicted GHs accounted for a small fraction (0.87%) in our composting community metatranscriptome, this was similarly observed by others who investigated various lignocellulytic communities such as rice-straw enriched compost (0.97%) [1], soil-contacting sugarcane bagasse (0.97%) [3], termite lumen (0.78%) [11], bovine rumen (0.78%) [10], and macropod foregut (0.71%) [14]. Amongst GHs, oligosaccharide-degrading enzymes from GH3 family were highly expressed in our study. These enzymes are fundamental in lignocellulolytic processes [5] and were abundant in other lignocellulolytic environments [40]. Mhuantong et al. explored the metagenome of a microbial community extracted from soil-contacting sugarcane bagasse [3]. Six out of the 10 most abundant GH families in the reported metagenome are amongst the most expressed GHs in each week of our metatranscriptome. Therefore, despite the different environments and techniques used, these communities have a reasonable level of similarity. Enzymes from auxiliary activity families, attributed to lignin oxidative modification and lytic polysaccharides degradation, accounted for a very small fraction (3.3%) of all predicted CAZymes in our metatranscriptome, supporting the lack of sugarcane bagasse lignin removal or structural changes in this polymer. This could be associated with a low relative abundance of fungi in our composting cultures, especially in early stages of the time course. Experimental design that uses liquid culturing of compost inoculum could have an effect on fungal growth and hence ligninolytic enzymes expression [41]. Recent studies showed that the composting conditions without liquid phase were preferable for CAZymes enrichment [42]. Other factors such as medium composition [43], temperature, agitation, and inoculum source could also play critical role for suppressing fungal growth.

Majority of CAZymes predicted in our studies had bacterial origin, similar in composition and structure to other studies [2, 3, 17, 40, 44]. Our community was dominated by a metabolically diverse Proteobacteria and Bacteroidetes. As observed previously, Proteobacteria dominates oxygenated habitats [3] and Bacteroidetes are known for their contribution to the largest reservoir of CAZymes in various environments [3, 40].

Interestingly, in the later stages of composting process, CAZymes expression shifted towards Eukaryotes and Animal kingdom. Representatives of nematodes, protists and other groups will be present in a composting spot, but it is unlikely that they would survive weeks in the submerged cultures. One explanation can be that the algorithm LCA did not assign phylogeny correctly. Also de novo assembly of metatranscriptomics reads and their mapping without reference genome can produce errors. However, in recent years, an increasing evidence of Eukaryotic invertebrates showed that their critical role in the hydrolysis of plant cell wall [45–48] and this aspect of our work should be further investigated.

Our comprehensive analysis led to identification of potentially, novel CAZy proteins. The recombinant expression efficiency in this work demonstrates the challenge that remains in characterizing novel genes derived from culture-independent approaches using heterologous systems. The solubility was confirmed for three target proteins (26%) but was lower than the 53% rate usually obtained in our laboratory using the same expression system [49]. A β-1,3-(4)-glucanase with specificity towards substrates with higher β-1,3 to β-1,4 ratio [50, 51], and no activity for CMC was found in our study. Compost7_GH6 was highly tolerant to an alkaline environment, an essential characteristic for application in detergent industry [52]. Compost13_GH10 presented substrate specificity and hydrolysis profile of a typical endoxylanase [53–55]. In contrast, the enzyme compost21_GH11 presented a hydrolysis profile of a typical exo-enzyme, releasing xylobiose from xylan and xylooligosaccharides. The structure of compost21_GH11 (Fig. 7b–d) shows a typical GH11 fold of a β-jelly-roll [56–60]. The architecture of other GH11 members shows the same pattern with little variation in the secondary structures lengths [56]. Despite 32 three-dimentional structures of GH11 members that have already been solved (http://www.cazy.org/GH11_structure.html), the compost21_GH11 structure reveals two extra loops previously unseen in the other family members. However, multiple alignment analysis revealed that there are many other proteins that might have these extra loops. Here, we show that loop EL2 blocks one side of compost21_GH11 active site, transforming this enzyme into an exo-1,4-β-xylanase that acts from the non-reducing end. To our knowledge, the present study describes the first example of an exo-xylanase from the GH11 family. compost21_GH11 has high activity on insoluble polymeric xylan, in contrast to GH8 exo-oligoxylanases that show preference for soluble xylooligosaccharides [61, 62].

It has been reported that xylooligosaccharides are strong cellulase inhibitors, whereas xylose and xylobiose have a smaller inhibitory effect [63]. As commercial enzymatic cocktails might have insufficient xylanase activity, a significant amount of xylooligomers accumulates in the reaction [20]. Hence, supplementation of enzyme cocktails with compost21_GH11 proved to increase their performance when there are xylooligomers in the reaction mixture. Therefore, in biomass treatment processes where xylooligomers accumulate [20], supplementing cocktails with compost21_GH11 will improve enzyme performance.

## Conclusions

In summary, our results indicate the ability of sugarcane bagasse adapted microbial community in deconstructing lignocellulosic biomass by removing the cellulose and hemicellulose fractions. The taxonomic binning and expression profile of GHs illustrate the degradation of lignocellulosic biomass complexity. Phylogenetic analysis also suggested a growing participation of eukaryotic microorganisms in this process, indicating that the organisms studied up to now may not represent the major organisms that degrade plant biomass in nature. Expression of genes selected from the metatranscriptome library revealed challenging. However, considering the industrially appealing features of proteins described here, we proved the importance of this line of study. The isolated enzymes warrant further study to characterize their structure and verify their ability to enhance commercially available cocktails, as have been proposed.

## Methods

### Sample collection and culture

Composting samples were collected from the São Paulo University Recycling Project (São Carlos campus) during the final mesophilic phase at locations 30 cm below the surface. A 1% (w/v) homogenized composting sample was used to inoculate minimal medium [64]. Cultures were supplemented with 3% (w/v) sugarcane bagasse and incubated at 30 °C with 150 rpm agitation for up to 5 weeks. Sugarcane bagasse was kindly provided by the Cosan Group (Ibaté, São Paulo, Brazil) and prior to use, it was washed and dried at 50 °C. Weekly sampling was performed on three biological replicates. Sugarcane bagasse and microbial biomass were separated from culture supernatant by centrifugation at 3000×*g* for 5 min at room temperature and used for nucleic acid extraction and biomass analysis.

### Biomass analysis

Prior analysis, sugarcane bagasse obtained from composting cultures was washed, dried, and ground to a fine powder using ball milling (TissueLyser II, Qiagen, Hilden, Germany) for 60 s at 30 Hz. The raw sugarcane bagasse was used as a control.

NMR experiments were performed using a Bruker Avance 400 spectrometer, equipped with a Bruker 4-mm magical angle spinning double-resonance probe, at ^13^C and ^1^H frequencies of 100.5 and 400.0 MHz, respectively. The spinning frequency at 14 kHz was controlled by a pneumatic system that ensures a rotation stability higher than ~ 1 Hz. Typical *π*/2 pulse lengths of 4 and 3.5 µs were applied for ^13^C and ^1^H, respectively. Proton decoupling field strength of γB_1_/2*π* = 100 kHz was used. ^13^C quantitative spectra were measured by using the Multi-CP excitation method described by Johnson and Schmidt-Rohr [21]. A total of nine cross polarization blocks were implemented with 1 ms and RF amplitude increment (90–100%), while the cross polarization before acquisition was executed with 0.8 ms and the same amplitude increment. The recycle delay was 2 s and the duration of the repolarization period *t*_*z*_ was 0.9 s [22]. To obtain the fraction of cellulose, hemicellulose and lignin, the spectra were normalized with respect to their area and integrated over the specific regions for cellulose (1 and 1′), lignin (2 and 2′) and hemicellulose (3 and 3′). The relative percentage of each component was obtained by dividing the calculated value for the biomass after microbial growth with the value obtained in the control sample. Finally, this fraction was multiplied by the initial percentage of the component in the raw sugarcane bagasse, as reported by Lima et al. [32]. Chemical shifts were assigned based on published studies [23–31]. Approximately 4000 scans were measured to acquire each spectrum. Chemical shift was assigned based on published studies [23–31].

Confocal microscopy was performed using a Zeiss LSM 780 confocal inverted microscope with a Coherent Chameleon laser (Ti:sapphire) as source for two-photons (2P) excitation at 800 nm. Ground sugarcane bagasse was hydrated for 24 h and observed with a C-Apochromat objective lens (20 ×, numerical aperture 0.8); the images were taken in the opposite side of the cover slip. The images were obtained by the average of 2 scans and no appreciate variation was observed. The spatial resolution was approximately 350 nm (considering the numerical aperture and the wavelength of excitation).

Scanning electron microscopy was performed using a scanning electron microscope model JSM-6390 LV (Jeol, Tokyo, Japan) operating with a 5 kV accelerating voltage. Ground sugarcane bagasse was hydrated for 24 h prior analysis; a drop was directly applied to the sample pedestal and dried at room temperature for 12 h. After drying, samples were gold coated using a metalizer model MED 020 (Bal-tec, Liechtenstein). Images were obtained under vacuum. At least 10 images per sample were acquired from different areas to certify the reproducibility of the results.

### Nucleic acid extraction from sugarcane composting cultures

A culture pellet (0.5 g of sugarcane bagasse and microbial cells) was used for cell lysis and nucleic acid extraction following a protocol modified from Griffiths et al. [65]. Briefly, 0.5 g pellet was added to 2-mL screw-cap tubes containing 0.5 g of acid washed 0.1-mm glass and 0.5-mm silica beads (each). 500 μL of CTAB extraction buffer (10% w/v hexadecyltrimethylammonium bromide in 700 mM NaCl mixed with an equal volume of 240 mM potassium phosphate buffer, pH 8.0) and 500 μL of phenol:chloroform:isoamyl alcohol (25:24:1) (pH 8.0) were added. Samples were lysed in a Bead Ruptor 24 (Omni, Kennesaw, GA, USA) for 30 s at 5.5 m/s and centrifuged at 17,000×*g* for 5 min at 4 °C. The top aqueous phase was transferred to a new tube and extracted with an equal volume of chloroform:isoamyl alcohol (24:1) followed by centrifugation at 17,000×*g* for 5 min at 4 °C. Total nucleic acid was precipitated with two volumes of PEG solution (30% w/v polyethylene glycol 6000 with 1.6 M NaCl) for 2 h at room temperature. Pellet was obtained by centrifuging the solution at 17,000×*g* for 20 min at 4 °C and washed twice with 70% ethanol. The nucleic acids were suspended in 50 μL of water and stored at − 80 °C until use. Its quality was determined with a LabChip GXII (PerkinElmer, Waltham, MA, USA).

All solutions and glassware were treated with 0.1% DEPC overnight at 37 °C under homogenization and autoclaved to create an RNase-free environment. Only certified RNase- and DNase-free plasticware was used.

### Real-time PCR of isolated genomic DNA

The nucleic acid extracted from composting cultures was diluted to 200 ng/μL and treated with 1:100 (v/v) RNase A:nucleic acid (Thermo Fisher Scientific, Waltham, MA, USA) for 15 min at 37 °C. The metagenomic DNA was extracted with phenol:chloroform:isoamyl alcohol and precipitated with 1/10 volume of 3 M sodium acetate pH 5.2 and 3 volumes of ethanol. Samples were incubated at 4 °C for 30 min and centrifuged at 17,000×*g* for 30 min at 4 °C. Supernatant was discarded. The isolated metagenomic DNA was washed with 70% ethanol and suspended with water.

Real-time PCR was performed using the metagenomic DNA and universal primer sets for bacterial (515F and 806R) [66] and fungal (ITS1 and 5.8S) [67] rDNA. PCR reactions contained 20-μL mixture of the following: 2.5 ng DNA, 300 nM of each forward and reverse primer, and 10 μL of KAPA SYBR^®^ FAST qPCR Master Mix (KAPA Biosystems, Wilmington, MA, USA), which contained all the nucleotide, polymerase, reaction buffer, and SYBR green dye. The thermocycling conditions were as follows: an initial hold at 95 °C for 5 min followed by 35 cycles of 95 °C for 30 s and 60 °C for 45 s, according to KAPA Biosystems recommendation. Measurements were done using a CFX96 Real-Time System (Bio-Rad, Hercules, CA, USA). All reactions were performed in triplicate during two independent experiments.

### cDNA library synthesis and sequencing

The nucleic acid extracted previously was diluted to 200 ng/μL and treated with DNase I (Invitrogen, Waltham, MA, USA) according to manufacturer’s recommendation. Equimolar volumes of the extracted RNA from biological replicates were combined, and the Prokaryotic ribosomal RNA (rRNA) was depleted with RiboZero Magnetic Kit Bacteria (Epicentre). The remaining RNA was purified using the RNA Clean & Concentrator-5 kit (ZymoResearch, Irvine, CA, USA). TruSeq Stranded Total RNA Sample Preparation kit (Illumina, San Diego, CA, USA) was used to deplete Eukaryotic rRNA and to synthesize a ~ 450 bp cDNA library. Sequencing of each time point cDNA library was performed on a MiSeq with a 500-cycle Reagent kit v2 (Illumina, San Diego, CA, USA).

### Metatranscriptomics assembly and annotation

Sequenced reads were preprocessed with Trimmomatic [68] to remove adaptors, low quality and short sequences. SortMeRNA [69] was used to merge and remove contaminant ribosomal RNA sequences, which were identified using Silva [70] and Rfam [71] reference databases with an e-value cutoff of 1 × 10^−5^. Non-ribosomal RNA reads were de novo assembled with Trinity [72] and genes were predicted using TransGeneScan [73]. Expression levels were calculated with eXpress [74] and Bowtie2 [75]. The phylogenetic origin of predicted ORFs was analyzed using MEGAN v6 [76] and the Lowest Common Ancestor algorithm at default values. HTSeq [77] software was used to subsample the sequenced reads. Rarefaction curves were plotted using the software BBMap (sourceforge.net/projects/bbmap/) for sequencing depth analysis. Functional annotation was performed with HMMER alignment tool [34] against the dbCAN database [35]. Kyoto Encyclopedia of Genes and the Genomes (KEGG) [78] orthology classification was performed using the online tool GhostKOALA [79] and default values.

### Target genes cloning, expression, and purification

Twenty-seven predicted CAZymes were selected for expression studies. The genes were codon optimized for *Escherichia coli* expression (https://www.idtdna.com/CodonOpt) and synthesized (GenScript, Jiangsu, China) after the predicted signal peptide and transmembrane helix was removed. Additionally, adapters were added to the 5′ (CAGGGCGCCATG) and 3′ (TAACCGCGTCGGGTC) sequence ends to allow cloning using ligation independent cloning (LIC) [80]. Standard molecular biology techniques were applied [81]. The gene fragments were cloned to pETTRXA-1a/LIC plasmid [49] and transformed into *E. coli* Rosetta (DE3) pLys cells (Merck, Darmstadt, Germany). Small-scale protein expression and solubility assays were performed as described previously [49]. Recombinant cells were stored at − 80 °C in the presence of 20% (v/v) glycerol.

Cells were grown overnight in the LB medium in the presence of kanamycin (50 μg/mL) and chloramphenicol (34 μg/mL) at 150 rpm shaking at 37 °C. 1 L of LB medium was inoculated with overnight culture (1% v/v) and incubated under 150 rpm shaking at 37 °C until the optical density at 600 nm reached 0.8. Incubation temperature was decreased to 17 °C and expression was carried out for 16 h after induction with 0.5 mM IPTG. The cells were harvested at 9000×*g* for 20 min and resulting pellet was resuspended in 20 mL of lysis buffer (20 mM Tris–HCl, 300 mM NaCl, 5 mM imidazole, 5% (v/v) glycerol, 10 mM β-mercaptoethanol (β-ME), 1 mM phenylmethylsulfonylfluoride (PMSF), 0.2 mg/mL lysozyme, pH 8.0) with or without 0.6% (w/v) sarkosyl. Cells suspension was incubated on ice for 1 h, sonicated for 6 min and centrifuged at 23,000×*g* for 30 min. Supernatant was loaded on a column with 2 mL of nickel-nitrilotriacetic acid (Ni–NTA) resin (Qiagen, Hilden, Germany) previously equilibrated with 10 volumes of lysis buffer. The column was washed with 4 volumes of wash buffer (20 mM Tris–HCl, 300 mM NaCl, 5 mM imidazole, 5% (v/v) glycerol, 10 mM β-ME, 1 mM PMSF pH 8.0) and in-column digestion was performed by adding cleavage buffer (20 mM Tris–HCl, 300 mM NaCl, 5% (v/v) glycerol, 10 mM β-ME, 1 mM PMSF, pH 8.0) and 1:50 TEV:protein (measured by 280 nm absorbance). After overnight incubation at 10 °C with homogenization, the recombinant protein was eluted in the flow-through. The column was further washed with 4 volumes of elution buffer (20 mM Tris–HCl, 300 mM NaCl, 300 mM imidazole, 5% (v/v) glycerol, 10 mM β-ME, 1 mM PMSF, pH 8.0). The protein was further purified using Superdex™ 75 16/60 (GE Healthcare Biosciences Corporation, Picataway, USA) column previously equilibrated with 20 mM Tris–HCl, 200 mM NaCl, pH 8.0. The protein purity was determined by sodium dodecyl sulfate–polyacrylamide gel electrophoresis (SDS-PAGE) and Coomassie blue staining [82].

### Sequence analysis and enzyme characterization

Multiple alignment of amino acid sequence was performed with Clustal Omega (http://www.ebi.ac.uk/Tools/msa/clustalo) [83]. Enzyme activity was determined by the amount of reducing sugars released from polysaccharide (Megazyme, Ireland; Sigma-Aldrich, St. Louis, MO, USA) using the DNS method [84]. Xylan and other substrates (see Additional file 1: Figure S3c) were used at 1 and 0.5% (w/v) final concentration, respectively. Glucose was used as a standard. All assays were performed in quadruplicate. Initial activity assays were performed at mild conditions using an array of substrates. Further enzyme assay was run at pHs ranging from 2 to 10 in 20 mM ABF buffer (20 mM of each sodium acetate, sodium borate and sodium phosphate dibasic; pH adjusted with HCl/NaOH) using optimal substrate. The reaction temperature was screened from 30 to 85 °C using the optimal substrate and pH. Finally, activity was screened against different polysaccharides at enzyme optimal pH and temperature conditions. Residual activity was tested by incubating the enzyme at optimal buffer pH for up to 48 h. Aliquots were removed and activity assays at optimal conditions were performed.

### Thermal stability analysis using ThermoFluor

To investigate the effect of pH on thermal stability, the protein was mixed with Sypro Orange (Invitrogen, Waltham, MA, USA), a reporter dye that binds nonspecifically to hydrophobic regions of the protein. Because water quenches the fluorescence of this dye, the fluorescence signal increases after the protein unfolds, allowing to monitor the melting curve. The experiment was performed on a CFX96 Real-Time System (Bio-Rad, Hercules, CA, USA) with excitation and emission wavelengths of 490 and 530 nm, respectively. 20 μL reactions were prepared with 0.2 mg/mL enzyme in different buffer solutions and 2000 times diluted dye. This mixture was added to a 96-well thin wall PCR plate (Bio-Rad, Hercules, CA, USA) and sealed with optical-quality sealing tape (Bio-Rad, Hercules, CA, USA). All buffers used in the analysis were prepared at 50 mM. The temperature scan was from 25 to 90 °C, with stepwise increments of 1 °C per minute. The melting temperature determination and analysis were performed using GraphPad Prism software v5.0 (GraphPad Software, La Jolla, CA, USA).

### Identification of enzymatic product on TLC and Dionex HPLC

The enzymatic reaction products were analyzed by TLC on silica gel 60 F254 (Merck, Darmstadt, Germany) with *n*-butanol:acetic acid:water (2:1:1, v/v) as eluent. The plates were developed with exposure to 10% (v/v) sulfuric acid in ethanol followed by charring. A mixture of xylooligosaccharides with 2 to 6 xylose residues (*X*2–*X*6) and xylose (*X*1) was used as standard.

Reaction products were also analyzed on a High-Performance Anion-Exchange chromatography with Pulsed Amperometric Detection (HPAE-PAD). The experiment was performed on a Dionex ICS-5000 Ion Chromatography system equipped with an electrochemical detector, a CarboPac PA1 (4 × 250 mm) anion exchange column and guard cartridge (Thermo Fisher Scientific, Waltham, MA, USA). The following program was used: flow 1 mL/min, 30 °C, isocratic 100 mM NaOH, [segment 1] 5–20′ from 0 to 20 mM CH_3_COONa, [segment 2] 20–24′ up to 100 mM CH_3_COONa, [segment 3] 24–30′ isocratic 100 mM CH_3_COONa.

### Xylooligosaccharide production and quantification

Xylooligosaccharides were produced following a protocol modified from Qing et al. [63]. Briefly, 5% (w/v) birchwood xylan was sealed in a 0.15-L stainless steel reactor. The reactor was transferred to a sand bath at 330 °C. After the temperature reached equilibrium at 200 °C, it was incubated for 10 min and quickly cooled in ice water. Solids were removed by centrifugation at 4000×*g* for 10 min and filtration through a 0.45-μm hydrophilic polyvinylidene fluoride (PVDF) filter (Merck, Kenilworth, NJ, USA). Dionex HPLC was employed to verify the xylooligomer distribution. The xylooligosaccharides were hydrolyzed in 4% (v/v) sulfuric acid for 1 h at 121 °C based on standard protocol from the National Renewable Energy Laboratory (NREL) [85]. Total oligomer concentration was determined using a HPLC (Shimadzu LC-20AT, Kyoto, Japan) equipped with refractive index and UV–VIS detectors and an aminex HPX-87H column (Bio-Rad Laboratories, Hercules, CA). Xylose standards were treated in parallel and used to calculate the sample concentration. The program used was as follows: flow rate 0.6 mL/min, 65 °C, isocratic 5 mM H_2_SO_4_.

### Xylooligosaccharide hydrolysis inhibition

2% (w/v) Avicel hydrolysis by Accellerase 1500 (DuPont, Wilmington, DE, USA) was performed in 50 mM sodium citrate pH 5.0 to access xylooligosaccharides inhibition (at 8 g/L) and activity improvement by compost21_GH11 addition (at 0.1 g/L). 0.02% (w/v) sodium azide was used to prevent microbial growth. Reactions were started by addition of Accellerase 1500 (DuPont, Wilmington, DE, USA) and Novozyme 188 (Sigma-Aldrich, St. Louis, MO, USA) diluted 25 and 250 times to give 5 FPU/g and 10 CBU/g, respectively. The flasks were incubated at 35 °C with 150 rpm agitation. Substrate blanks without enzyme and enzyme blanks without substrate were also set. 0.5 mL aliquots taken after 1, 4, 7, 24, 48, 72, and 96 h of hydrolysis were immediately boiled for 10 min to inactivate enzymes, centrifuged at 10,000×*g* for 1 min, filtered through a 0.45 μm PVDF filter and frozen at − 20 °C. Two independent experiments were performed. The cellulose conversion yields were analyzed with HPLC, as discussed before, using glucose standards. This experimental setup was based on NREL standard protocol [86].

### Crystallization and data collection

Crystallization conditions were screened for crystal growth using a HoneyBee crystallization robot 931 (Genomic Solutions, Ann Arbor, MI, USA) and commercial available screens. Crystals were obtained for protein compost21_GH11 in multiple conditions. Crystals grown at 18 °C in 0.1 M BIS–TRIS propane pH 7.5, 20% (w/v) PEG 3350, 0.2 M NaI were briefly soaked in a cryoprotective solution (crystallization solution with 15% (v/v) ethylene glycol added) and flash-cooled in a gaseous nitrogen steam at 100 K. The diffraction data were collected at the MX2 beamline [87] of the Brazilian National Synchrotron Laboratory (LNLS, Campinas, Brazil) using synchrotron radiation with wavelength set to 1.459 Å, PILATUS2 M detector (Dectris, Taefernweg, Switzerland) and an oscillation of 0.5° per frame. Diffraction data were reduced and integrated with XDS [88].

### Molecular replacement, model building, and structure refinement

The molecular replacement, structure model building, refinement, and validation were performed using PHASER [89], PHENIX [90], Coot [91], and MolProbity program [92]. PDB ID 1XNK was used as template. PyMOL (the PyMOL Molecular Graphics System, Version 1.8 Schrödinger, LLC) was used for structure representations.

## Additional file

**Additional file 1.** Additional figures and tables.

## Abbreviations

CAZyme: carbohydrate active enzyme
CMC: carboxymethyl cellulose
EL: extra loop
FPKM: fragments per kilobase of transcript per million
GH: glycoside hydrolase
KEGG: Kyoto Encyclopedia of Genes and the Genomes
Multi-CP: multiple cross polarization pulse sequence
ORF: open reading frame
ssNMR: solid-state nuclear magnetic resonance
TLC: thin layer chromatography
