# Supplementary material for: Targeted metatranscriptomics of compost-derived consortia reveals a GH11 exerting an unusual exo-1,4-β-xylanase activity
Source: Biotechnol Biofuels. 2017 Nov 2;10:254. doi: 10.1186/s13068-017-0944-4 (PMC5667448; doi:10.1186/s13068-017-0944-4)
Supplement: Supplementary file 1 — Additional file 1. Additional figures and tables. [file 13068_2017_944_MOESM1_ESM.docx]

**Supplementary Information**

**Figure legends**

**Fig. S1**

Rarefaction analysis of metatranscriptome sequencing depth from sugarcane bagasse composting community by two methods. The first is based on the assumption that the sequencing depth affects the statistics. Therefore, when sequencing becomes redundant, the statistics will be stable. The second method extracts k-mers from each read and checks if it has been seen before. For each 25,000 reads, a point is plotted with the percentage of new reads versus the number of reads processed. The sequencing is saturated after zero is reached. **a** The predicted expression level using the entire and rarefactioned libraries were compared at different sequencing depths. At 90% rarefaction, most of the genes have less than 10% fragments per kilobase of transcript per million (FPKM) relative error, but there are still genes with more than 90% relative error. **b** Percentage of unique k-mers as more reads are sequenced. Based on both methods, the sequencing saturation was not reached.

**Fig. S2**

Phylogenetic assignment of the expressed CAZymes in sugarcane bagasse composting community through time using the Lowest Common Ancestor algorithm. **a** Relative expression of bacterial phyla. The abundance of genes assigned to Bacteroidetes showed an increase from 29% to 44% during 5-week trail, in contrast to genes originating from Proteobacteria that showed opposite trend by decreasing from 42% to 24%. The phylum Firmicutes showed a gradual increase from 1% to 5%. **b** Eukaryotic kingdoms. The expression of CAZymes from non-fungal kingdoms highly grew over time. The total expression of each domain is represented by the gray line.

**Fig. S3**

Biochemical characterization of the compost7_GH6, compost13_GH10 and compost21_GH11 proteins derived from sugarcane bagasse composting community. Effect of **a** pH and **b** temperature on enzyme activity. **c** Substrate specificity examined towards an array of polysaccharides. **d** Residual activity after incubation in the studied temperature.

**Fig. S4**

Thermal stability of compost7_GH6 protein examined at different pH values as assessed by ThermoFluor.

**Tables**

**Table S1**

| **Growth weeks** | **Relative percentage of fungi to bacteria rDNA** |
| --- | --- |
| **0** | 11 ± 2 |
| **1** | 4.8 ± 0.4 |
| **2** | 9 ± 1 |
| **3** | 21 ± 3 |
| **4** | 20 ± 3 |
| **5** | 22 ± 1 |

Relative abundance of rDNA amplified from fungal and bacterial specific regions.

**Table S2**

| **ID** | **Length (AA)** | **Completeness** | **e-value** | **Alignment parameters with best hit** | | | | **Expression** | **Characterized** |
| --- | --- | --- | --- | --- | --- | --- | --- | --- | --- |
|  |  |  |  | **Length** | **Identity/Gap (%)** | **Accession number** | **Taxonomy** |  |  |
| **Compost 1_GH5** | 326 | 3' partial | 0.0 | 326 | 52 / 1 | gi\|919149142 | *Teredinibacter sp.* | N | - |
| **Compost 2_GH5** | 239 | internal | 3.2E-08 | 233 | 37 / 1 | gi\|775268352 | *Acidisphaera rubrifaciens* | N | - |
| **Compost 3_GH5** | 325 | 5' partial | 6.4E-24 | 341 | 47 / 7 | gi\|737251030 | *Acidobacteriaceae bacterium* | N | - |
| **Compost 4_GH5** | 366 | 5' partial | 4.7E-22 | 330 | 48 / 1 | gi\|931376366 | *Coxiella sp.* | N | - |
| **Compost 5_GH5_5** | 355 | 5' partial | 5.1E-24 | 335 | 43 / 2 | gi\|931376366 | *Coxiella sp.* | N | - |
| **Compost 6_GH6** | 284 | 3' partial | 7.7E-30 | 264 | 77 / 1 | gi\|653077963 | *Marinimicrobium agarilyticum* | Y | N |
| **Compost 7_GH6** | 390 | 5' partial | 0.0 | 373 | 49 / 3 | gi\|1005329896 | *Sorangium cellulosum* | Y | Y |
| **Compost 8_GH6** | 273 | 3' partial | 2.2E-20 | 246 | 74 / 1 | gi\|653077963 | *Marinimicrobium agarilyticum* | Y | N |
| **Compost 9_GH6** | 324 | internal | 0.0 | 326 | 48 / 3 | gi\|546309190 | *Chondrus crispus* | Y | N |
| **Compost 10_GH6_5** | 377 | internal | 0.0 | 380 | 48 / 1 | gi\|546309190 | *Chondrus crispus* | N | - |
| **Compost 11_GH7** | 445 | 5' partial | 0.0 | 438 | 68 / 0 | gi\|761948412 | *Cylindrobasidium torrendii* | N | - |
| **Compost 12_GH9** | 514 | 5' partial | 7.3E-28 | 456 | 49 / 2 | gi\|797005938 | *Teredinibacter sp.* | N | - |
| **Compost 13_GH10** | 287 | 5' partial | 0.0 | 285 | 91 / 0 | gi\|769243366 | *Sorangium cellulosum* | Y | Y |
| **Compost 14_GH10** | 334 | 5' partial | 0.0 | 327 | 95 / 0 | gi\|1005175543 | *Sorangium cellulosum* | N | - |
| **Compost 15_GH10** | 274 | complete | 2.0E-44 | 269 | 50 / 5 | gi\|797008181 | *Teredinibacter sp.* | Y | N |
| **Compost 16_GH10** | 306 | internal | 0.0 | 295 | 38 / 8 | gi\|1310760 | *Clostridium thermocellum* | N | - |
| **Compost 17_GH10_5** | 258 | internal | 0.0 | 264 | 52 / 4 | gi\|161162172 | *Sorangium cellulosum* | N | - |
| **Compost 18_GH11** | 253 | complete | 0.0 | 256 | 78 / 2 | gi\|902716143 | *Cellvibrio sp.* | N | - |
| **Compost 19_GH11** | 244 | complete | 0.0 | 239 | 85 / 0 | gi\|902716143 | *Cellvibrio sp.* | N | - |
| **Compost 20_GH11** | 183 | 5' partial | 1.4E-31 | 184 | 38 / 5 | gi\|595588127 | *Neocallimastix patriciarum* | N | - |
| **Compost 21_GH11** | 227 | internal | 9.8E-45 | 229 | 77 / 0 | gi\|653077723 | *Marinimicrobium agarilyticum* | Y | Y |
| **Compost 22_GH12** | 263 | 5' partial | 6.3E-18 | 269 | 25 / 21 | gi\|496168814 | *Haloterrigena salina* | N | - |
| **Compost 23_GH12** | 250 | complete | 1.1E-11 | 364 | 29 / 43 | gi\|797011013 | *Teredinibacter sp.* | N | - |
| **Compost 24_GH12** | 203 | internal | 1.1E-18 | 162 | 27 / 30 | gi\|493937532 | *Halosimplex carlsbadense* | N | - |
| **Compost 25_GH45** | 310 | 5' partial | 0.0 | 241 | 46 / 6 | gi\|121816 | *Cellvibrio japonicus* | N | - |
| **Compost 26_GH45** | 200 | 5' partial | 0.0 | 222 | 49 / 10 | gi\|665990613 | *Alteromonadaceae bacterium* | N | - |
| **Compost 27_GH48** | 449 | internal | 0.0 | 452 | 96 / 0 | gi\|502883342 | *Cellulomonas flavigena* | N | - |

Parameters of the 27 targets selected for cloning. Some targets had one or both ends missing during sequencing/assembly. However, the predicted domain was fully present. The genes expressed in *E. coli* soluble fraction that were successfully characterized are highlighted.
